# PERSONALITY TRAITS PREDICT DIABETES RISK IN ADULTHOOD: THE MEDIATING EFFECTS OF USING FOOD TO COPE WITH STRESS

**DOI:** 10.1093/geroni/igac059.1465

**Published:** 2022-12-20

**Authors:** Nicholas Turiano, Jacob Alderson, Meredith Willard, Sina King, Páraic Ó Súilleabháin

**Affiliations:** West Virginia University, Morgantown, West Virginia, United States; West Virginia University, Morgantown, West Virginia, United States; West Virginia University, Morgantown, West Virginia, United States; West Virginia University, Morgantown, West Virginia, United States; University of Limerick, Limerick, Limerick, Ireland

## Abstract

Identifying the individual characteristics that predict which adults will develop obesity and diabetes is crucial. This study included national data from 902 participants (aged 25-75) in the Midlife Development in the U.S. (MIDUS) study. Participants completed the Big-5 personality trait measure in 1995-1996, and behavior/health variables between 2004-2009. We tested whether levels of certain personality traits would predict an elevated risk of diabetes via hemoglobin A1c (HbA1c) levels through eating behaviors. A structural equation modeling framework demonstrated good fit when testing indirect effects (CFI = 0.95; RMSEA = 0.05). Indirect effects revealed that higher levels of neuroticism predicted greater waist circumference and higher HbA1c levels due to an increased use of food to cope with problems (IE =0.10; p<0.05). Moreover, indirect effects were found for conscientiousness, albeit in a protective direction. Our findings suggest that personality traits may be an early predictor of behavior and thus long-term adverse health outcomes.

